# Migraine severity, disability, and duration: Is a good diet quality, high intake of phytochemicals and polyphenols important?

**DOI:** 10.3389/fnut.2022.1041907

**Published:** 2022-11-21

**Authors:** Hande Bakırhan, Merve Pehlivan, Tuğba Uyar Cankay, Mehmet Kocak

**Affiliations:** ^1^Department of Nutrition and Dietetics, Faculty of Health Sciences, Istanbul Medipol University, Istanbul, Turkey; ^2^Department of Nutrition and Dietetics, Institute of Health Sciences, Istanbul Medipol University, Istanbul, Turkey; ^3^Department of Neurology, Medeniyet University Göztepe Training and Research Hospital, Istanbul, Turkey; ^4^Biostatistics and Bioinformatics Analysis Unit, Division of Biostatistics and Medical Informatics, International School of Medicine, Istanbul Medipol University, Istanbul, Turkey

**Keywords:** diet quality, polyphenol, phytochemicals, migraine, headache

## Abstract

**Objectives:**

Dietary pattern may be the determinant of migraine prognosis through various mechanisms such as systemic inflammation, vasodilation, cerebral glucose metabolism, and mitochondrial dysfunction.

This study was conducted to examine the relationship of the symptoms and signs of migraine with dietary polyphenols and the phytochemical intake and the quality of the diet.

**Materials and methods:**

Individuals (*n* = 90), who were admitted to the headache outpatient clinic due to the diagnosis of episodic migraine, underwent physical examination by a neurologist. Migraine characteristics were assessed using the Migraine Disability Assessment Questionnaire and the Visual Analogue Scale. The Healthy Eating Index-2015 (HEI-2015) was used to evaluate the diet quality of individuals, and the Phytochemical Index developed by McCarty was used to determine the dietary intake of phytochemicals. Phenol-Explorer version 3.6 and the USDA Database for the Flavonoid Content of Selected Foods-Release 3.3 were used to calculate the dietary polyphenol intake.

**Results:**

Migraine severity was negatively correlated with the intake of phytochemicals and good diet quality (*r* = −0.37, *p* = 0.0003; *r* = −0.37, *p* = 0.0003, respectively), and with the intake of phenolic components flavanones (*r* = −0.27, *p* = 0.01) and lignans (*r* = −0.27, *p* = 0.01). With respect to the food groups; migraine severity was found to be inversely correlated with the total phenol intake from olive oil, oil, and fruits (*r* = −0.26, *p* = 0.01; *r* = −0.21, *p* = 0.04; *r* = −0.24, *p* = 0.02, respectively), and the flavonoid intake from olive oil, oil, fruits, and vegetables (*r* = −0.26, *p* = 0.01; *r* = −0.26, *p* = 0.01; *r* = −0.35, *p* = 0.0007; *r* = −0.22, *p* = 0.04, respectively). Strikingly, fruit flavanone intake was correlated with low migraine severity (*r* = −0.39, *p* = 0.0002), and fruit flavanol intake was correlated with low migraine disability (*r* = −0.21, *p* = 0.04).

**Conclusion:**

A high-quality diet rich in phytochemicals and polyphenols (especially flavanones and lignans) is associated with low migraine severity. Lower intake of phenols and flavonoids from vegetable oil, olive oil, fruits, and vegetables were associated with more severe migraine attacks. Examination of migraine characteristics and dietary pattern together with phytochemical and polyphenol intake may guide the development of dietary strategies to be used in migraine patients.

## Introduction

Migraine is a type of headache, which is characterized by throbbing pain, recurring attacks, and significant reductions in the quality of life (1–3). In the presence of unknowns in the pathophysiology of migraine, several vascular, neurogenic, biochemical, and thrombocytic theories have been proposed to explain the pathogenesis (4, 5). The frequency and development of migraine attacks are regulated by the central nervous system, in a manner sensitive to environmental factors. Among other characteristics, the onset of migraine attacks is associated with trigger factors (6). The trigger factors of migraine can be examined in two groups as non-nutritional and nutritional factors. Foods containing monosodium glutamate, nitrites (meat with added preservatives, processed meat products), tyramine (wine, cheese), and phenylethylamine (chocolate, garlic, onion, and nuts) are potential trigger factors. Foods including alcohol, sweeteners, citrus fruits, pickles, and vinegar are potential causative factors triggering migraine. Trigger foods are variable and specific to individuals. The elimination of such foods from the diet does not necessarily mean freedom from migraine attacks (3). The actions of nutritional factors on the nervous system originate from their ability to affect nerve density and various nerve-related changes. It has been reported that foods can induce pain by causing contraction and expansion of neurons (7). Dietary factors may be predictors of migraine prognosis because of their involvement in systemic inflammation, vasodilation, cerebral glucose metabolism, mitochondrial dysfunction, and the frequency and severity of migraine attacks (8).

With the suggested important role of oxidative stress in the pathogenesis of migraine, nutraceuticals, including antioxidants, come to the fore among substances that can potentially be used for migraine treatment. Antioxidants from food can prevent oxidative stress by inhibiting a series of oxidative chain reactions. Substances with an antioxidant composition including carotenoids (xanthophylls and carotenes) and polyphenols (phenolic acid, anthocyanins, lignans, flavonoids, and phenolic acid) can help prevent migraines or alleviate symptoms through anti-oxidative effects, mitochondrial energy metabolism maintenance, anti-inflammatory and anti-apoptotic properties (9). Dietary patterns and diet quality can affect the duration, frequency, and severity of migraine headaches. Several studies have been conducted to examine the role of food intake and various dietary components in migraine (7, 10–13) but there are no studies on patients to investigate the association of the quality, polyphenol components, and phytochemical content of the diet with the signs and symptoms of migraine.

This study aims to holistically evaluate diet quality, polyphenol and phytochemical intake, and migraine symptoms. To our knowledge, this study is the first of its kind examining dietary phytochemical and polyphenol intake together with diet quality in migraine patients. The main aim of the study is to investigate the relationship between diet quality, polyphenol and phytochemical intake with migraine characteristics and may lead to the development of dietary strategies that can be used in clinical practise in migraine management.

## Materials and methods

### Study design and sample selection

This study was conducted on 90 patients aged 19–65 years, who were admitted to Göztepe Training and Research Hospital’s Neurology Outpatient Clinic due to the diagnosis of episodic migraine. The diagnosis of episodic migraine was made by a neurologist according to the International Classification of Headache Disorder-3 (ICHD-3) beta version criteria (14). A questionnaire form was administered to subjects by the researcher during face-to-face interviews. The questionnaire form consisted of questions about the demographics (age, gender, educational status, etc.) and the medical history (previous diagnoses, the use of medications, etc.) of subjects. The questionnaire also included scales for the evaluation of the signs and symptoms of migraine [the migraine disability scale (MIDAS), the visual analog scale (VAS), and a questionnaire for migraine duration and frequency], and other tools for the assessment of the diet quality and the calculation of the dietary phytochemical and polyphenol content (a food intake record of subjects).

**FIGURE 1:**
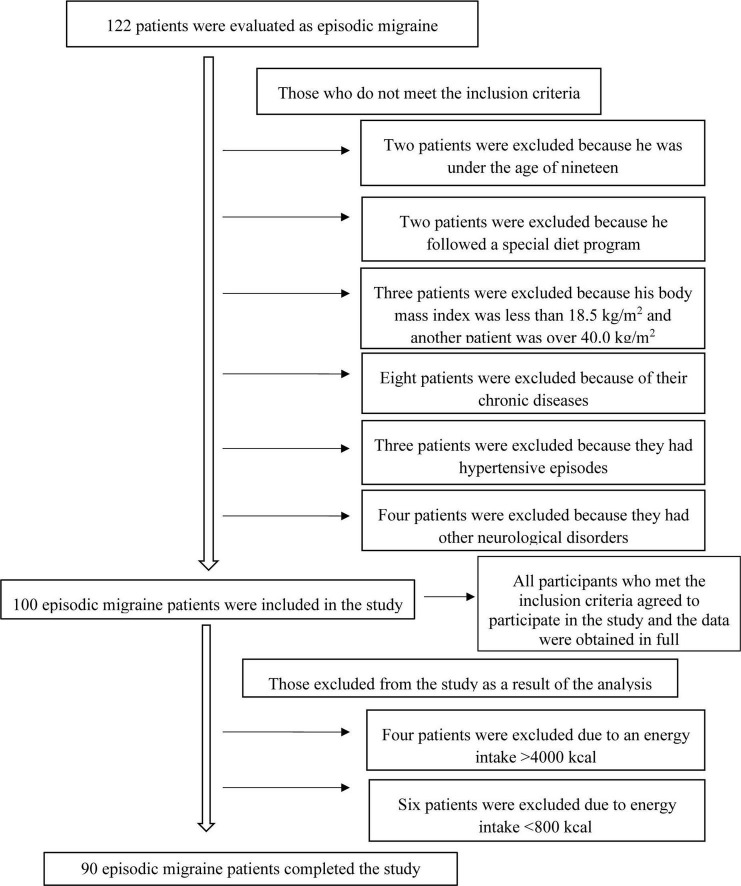
Patient recruitment flow chart.

Individuals were excluded, who had a body mass index of ≥40.0 kg/m^2^ or ≤18.5 kg/m^2^, who had chronic comorbidities (hypertension, cardiovascular diseases, diabetes, cancer, hepatic or renal diseases, or other neurological disorders, etc.), who used lipid-lowering medications or medications for glucose intolerance, who took vitamin-mineral supplements, who adopted a special diet, or who had a daily energy intake of fewer than 800 kilocalories or more than 4,000 kilocalories. Figure 1 shows the patient recruitment flowchart in line with the inclusion and exclusion criteria. The study protocol was approved by the Istanbul Medipol University Non-Interventional Clinical Research Ethics Committee on March 18, 2021, with decision number 303. All participants gave written informed consent. The study was conducted in accordance with the ethical principles of the Declaration of Helsinki.

### Assessment of migraine characteristics

A headache diary was filled out with each patient under the supervision of a neurologist during face-to-face interviews in order to assess the frequency, duration, and severity of the attacks over the last 3 months. MIDAS was used to assess the functional loss (disability) due to migraine, and VAS was used to assess migraine severity (15, 16). The mean severity of headaches over the last 3 months was inquired using the VAS scale. Patients stated the mean severity scores of their migraine headache attacks themselves. The severity of pain was categorized as mild for scores of ≤3, moderate for scores of 3–6, and severe for scores of >6 (15). Headache-related disability was evaluated with MIDAS to find the number of days lost due to migraine disability over the last 3 months based on patients’ statements. The degree of disability was categorized as none or very little for 0–5 days, mild for 6–10 days, moderate for 11–20 days, and severe for ≥21 days (16). The duration and frequency of migraine were recorded using a questionnaire.

### Assessment of dietary patterns

Diet quality was determined using food intake records taken from the participants. Data on the intake of nutrients were obtained prospectively through a 3-day non-consecutive food records. Participants were asked not to make any difference in their routine eating habits. How to fill in the food intake forms was explained to the participants by the researcher verbally and using visual materials (replicas, food catalogs) and food intake records were taken for three non-consecutive days, one of which was on the weekend. The food intake records were transferred to the “Computer-Assisted Dietary Assessment Software, a Diet Information System” (BeBiS, version 7) which is a food software program in compliance with Turkish food was used for assessment nutrients, food and food groups, to calculate the daily intake of energy and nutrients for each participant (17). The Healthy Eating Index-2015 (HEI-2015) was used to evaluate the diet quality of individuals, data obtained from the subjects’ food intake records were used to score the diet quality (18). Based on the total HEI-2015 scores, the diet quality of subjects was categorized as “poor” for scores of ≤50, “needs improvement” for scores of 51–80, and “good” for scores of >80 (18).

### Assessment of the phytochemical intake of the diet

The Phytochemical Index (PI) developed by McCarty was used to find the dietary phytochemical content of subjects. The PI is an index developed to correlate dietary phytochemical intake with health outcomes. It is a tool designed for clinical nutritionists to use to analyze diet quality, encourage consuming phytochemical-rich foods, and determine phytochemical intake level (19). The phytochemical index (PI) is calculated by dividing the energy intake from phytochemical-rich foods (kcal/day) by the total daily energy intake and multiplying the result by 100. Phytochemical-rich foods include fruits and vegetables (including tuber vegetables but excluding potatoes), oilseeds, nuts, whole grains, pulses, olives, and olive oil. Tomato sauces, vegetable juices, and natural fruits are included in the PI calculation because of their high phytochemical content. Tea, coffee, spices, and potatoes are not included in the PI due to their low phytochemical content (19).

### Calculation of the polyphenol intake of the diet

The daily intake (g/mL) of each eaten/drank food was calculated using the subjects’ food intake records. An advanced search was conducted on the Phenol-Explorer version 3.6 and the USDA Database for the Flavonoid Content of Selected Foods-Release 3.3 to find the average values of polyphenol content of each food and beverage in the food intake records of subjects. The polyphenol intake of each participant was calculated for each eaten or drank food (20).

The total (poly) phenol content was calculated for each selected compound class by adding up all individual phenolics obtained by chromatography without hydrolysis. When chromatographic values without hydrolysis were not available, all individual phenolics were added up using the obtained values by chromatography after hydrolysis. The total amount of phenolic compounds was calculated as the sum of individual phenol contents determined by the Folin assay method. In this study, we investigated exposure to total polyphenols and major polyphenol classes including phenolic acids, flavonoids, stilbenes, and lignans; the main subclasses of phenolic acids, including hydroxybenzoic acids, hydroxycinnamic acids, hydroxyphenylacetic acids, and hydroxyphenyl propanoic acids; the main subclasses of flavonoids, including flavanols, flavonols, flavanones, flavones, anthocyanins, dihydroflavonols, and isoflavones, and the category of “other” polyphenols. Some foods (strawberry jam, apricot jam, quinoa, chia, grape molasses, mulberry molasses, lemonade, Turkish coffee, rocket, pomegranate syrup, peas, pumpkin seeds, crackers, vine leaves, black tea, bulgur, rice, spring onions, chickpeas, and rye bread) in the food intake records were not included in the Phenol-Explorer version 3.6 database and the USDA Database for the Flavonoid Content of Selected Foods-Release 3.3. Information in the scientific literature was used in order to determine the polyphenol content in these foods.

The intake of foods and beverages was categorized according to the classification of foods in the Phenol-Explorer version 3.6 database. Alcoholic beverages were not included in the study because none of the participants used alcohol. Black tea, olive oil, and pulses are commonly eaten food in society, therefore, these were categorized as another class of foods. The polyphenol intake was examined in total and in the following subgroups including soft drinks, tea, cocoa and coffee, fruits, vegetables, oil, pulses, oilseeds, cereals, and olive oil.

### Statistical analysis of data

Categorical variables were reported as frequencies and percentages. Continuous variables were reported as mean and standard deviation. Associations between categorical variables were tested using the Chi-Square Test. The Cochran-Armitage Trend test was used to examine the significance of the correlation between a binary factor and an ordinal factor such as the tertiles of a marker to capture any potential trend. The distribution of a marker of interest was compared across the levels of a factor of interest by the Wilcoxon–Mann–Whitney or Kruskal–Wallis tests as the counterparts of the two-sample *t*-test and the one-way ANOVA, respectively. The correlation between dietary markers and migraine scores was estimated using Spearman’s Rank Correlation. Univariably significant variables were included in a stepwise variable selection process in multivariable regression models. The type-1 error rate was accepted as 0.05 and the results were provided without multiple-testing adjustment.

## Results

The distribution of migraine characteristics by the diet quality, phytochemical intake, and total polyphenol intake of participants is presented in Table 1. Of the participants, 82.7% with poor diet quality and 57.9% with good diet quality and with a need for improvement in the diet quality had migraines without aura (*p* < 0.05). Although the differences in the migraine attack frequency and related disability did not achieve significance by the diet quality, the polyphenol intake, and the phytochemical intake, the mean attack duration was significantly lower in participants with poor diet quality (17.7 ± 18.83 vs. 27.0 ± 21.83, *p* = 0.016) compared to others. The severity of migraine attacks was significantly higher in patients with poor diet quality than those with good diet quality and those needing improvements in their diet quality (7.6 ± 1.58 vs. 6.7 ± 2.36, *p* = 0.046). While 75.0% of participants with poor diet quality suffered from severe attacks, the rate of severe attacks was 47.4% for participants either having good diet quality or needing improvements in their diet quality (*p* = 0.0072). It was found that migraine severity decreased as phytochemical intake increased (VAS scores: 8.2 ± 1.42 vs. 6.2 ± 2.32, *p* = 0.001). While 86.7% of individuals with the lowest phytochemical intake (T1) suffered from severe migraine attacks, 60.0% of individuals with the highest intake (T3) had migraine attacks of mild or moderate severity (*p* = 0.0009).

**TABLE 1:** Participants’ migraine characteristics according to diet quality, phytochemical index, and total polyphenol intake.

	HEI-2015		Phytochemical index			Total phenol intake		
			
	*Poor*	*Good and needs improvement (%)*	*T1*	*T2*	*T3*	*T1*	*T2*	*T3*
	*(%)*		*n (%)*	*n (%)*	*n (%)*	*n (%)*	*n (%)*	*n (%)*
**Migraine type**								
With aura	9 (17.3)	16 (42.1)	5 (16.7)	9 (30.0)	11 (36.7)	5 (16.7)	10 (33.3)	10 (33.3)
Without aura	43 (82.7)	22 (57.9)	25 (83.3)	21 (70.0)	19 (63.3)	25 (83.3)	20 (66.7)	20 (66.7)
*P*-value	***0.0095***		*0.2121*				*0.2504*	
**Attack frequency**								
1–2 times a week	39 (75.0)	29 (76.3)	22 (73.3)	25 (83.3)	21 (70.0)	26 (6.7)	21 (70.0)	21 (70.0)
1–2 times a month	13 (25.0)	9 (23.7)	8 (26.7)	5 (16.7)	9 (30.0)	4 (13.3)	9 (30.0)	9 (30.0)
*P*-value	*0.8859*		*0.4575*				*0.222*	
**Attack duration**								
0–12 h	31 (59.6)	14 (36.8)	18 (60.0)	14 (46.7)	13 (43.3)	16 (53.3)	12 (40.0)	17 (56.7)
13–24 h	12 (23.1)	12 (31.6)	5 (16.7)	12 (40.0)	7 (23.3)	9 (30.0)	10 (33.3)	5 (16.7)
25–72 h	9 (17.3)	12 (31.6)	7 (23.3)	4 (13.3)	10 (33.3)	5 (16.7)	8 (26.7)	8 (26.7)
*P*-value	*0.0912*		*0.1494*				*0.4718*	
**Attack duration^a^**	17.7 ± 18.83	27.0 ± 21.83	19.2 ± 19.86	19.8 ± 18.80	25.9 ± 22.84	17.5 ± 16.46	24.0 ± 20.34	23.3 ± 24.23
*P*-value	***0.0165***		*0.6721*				*0.5469*	
**Use of migraine medication**								
No	48 (92.3)	31 (81.6)	26 (86.7)	28 (93.3)	26 (86.7)	27 (10.0)	26 (86.7)	27 (10.0)
Yes	4 (7.7)	7 (18.4)	4 (13.3)	2 (6.7)	4 (13.3)	3 (90.0)	4 (13.3)	3 (90.0)
*P*-value	*0.4065*		*0.6376*				*0.8936*	
**Migraine disability**								
None or very little and mild loss	19 (36.5)	14 (36.8)	9 (10.0)	15 (50.0)	9 (10.0)	9 (10.0)	12 (40.0)	3 (10.0)
Moderate loss and severe	33 (63.5)	24 (63.2)	21 (70.0)	15 (50.0)	21 (70.0)	21 (70.0)	18 (60.0)	27 (90.0)
*P*-value	*0.9764*		*0.1786*				*0.6501*	
**Migraine disability** (**MIDAS)^a^**	20.8 ± 20.01	26.7 ± 22.03	23.8 ± 23.14	19.2 ± 19.67	26.8 ± 19.91	24.7 ± 21.13	21.5 ± 21.30	23.7 ± 21.08
*P*-value	*0.2049*		*0.2260*				*0.8189*	
**Attack severity (VAS)**								
Mild and moderate	13 (25.0)	20 (52.6)	4 (13.3)	11 (36.7)	18 (60.0)	9 (30.0)	13 (43.3)	11 (36.7)
Severe	39 (75.0)	18 (47.4)	26 (86.7)	19 (63.3)	12 (40.0)	21 (70.0)	17 (56.7)	19 (63.3)
*P*-value	***0.0072***		***0.0009***				*0.5632*	
**Attack severity (VAS)^a^**	7.6 ± 1.58	6.7 ± 2.36	8.2 ± 1.42	7.4 ± 1.65	6.2 ± 2.32	7.3 ± 2.01	6.9 ± 1.99	7.5 ± 1.98
*P*-value	***0.0467***		***0.0010***				*0.5399*	

^a^Mean ± SD. Statistically significant values (*p* < 0.05) were given in bold. *P*-values are in italics.

The duration, severity, and associated disability of migraine attacks by the polyphenol intake levels of subjects are presented in Table 2. Migraine severity was lower in subjects with a moderate intake of polyphenols compared to the other groups of intake (6.9 ± 1.99 vs. 7.3 ± 2.01 and 7.5 ± 1.98, *p* = 0.048). It was observed that migraine severity significantly increased with the increased intake of phenolic components in soft drinks (6.7 ± 2.09 vs. 7.3 ± 1.70, *p* = 0.048). However, with the increased intake of phenolic components in fruits (7.9 ± 1.52 vs. 6.4 ± 2.33, *p* = 0.031), vegetables (7.7 ± 1.55 vs. 6.4 ± 2.33, *p* = 0.030), and olive oil (7.5 ± 1.75 vs. 6.2 ± 2.43, *p* = 0.012), migraine severity decreased significantly. In individuals with a moderate intake of phenolic components in tea, the severity of migraine attacks was lower but the difference was not statistically significant (6.7 ± 2.04 vs. 7.9 ± 2.06 and 7.3 ± 1.73, *p* = 0.055). With respect to migraine disability, it was observed that MIDAS scores increased significantly only with the increased intake of the phenolic component in pulses (*p* = 0.007).

**TABLE 2:** Migraine attack duration, severity, and disability according to participants’ polyphenol intake.

	MIDAS score	VAS score	Duration
	*Mean* ± *SD*	*Mean* ± *SD*	*Mean* ± *SD*
**Phenol**			
**Non-alcoholic beverages**			
T1	19.0 ± 18.59	6.7 ± 2.09	21.4 ± 17.11
T2	27.4 ± 20.53	7.3 ± 2.04	25.0 ± 22.28
T3	23.1 ± 23.18	7.3 ± 1.70	18.4 ± 21.73
*P*-value/χ^2^	*0.1752*	***0.0480***	*0.3440*
**Black tea**			
T1	18.5 ± 18.39	7.9 ± 2.06	20.7 ± 17.18
T2	27.4 ± 20.53	6.7 ± 2.04	25.0 ± 22.28
T3	23.6 ± 23.38	7.3 ± 1.73	19.0 ± 21.89
*P*-value/χ^2^	*0.1523*	*0.0555*	*0.4409*
**Coffee and cocoa**			
T1	23.9 ± 23.49	7.3 ± 2.02	21.2 ± 19.15
T2	24.6 ± 14.71	6.3 ± 1.86	37.0 ± 27.86
T3	21.9 ± 17.34	7.3 ± 1.96	19.2 ± 20.94
*P*-value/χ^2^	*0.8469*	*0.2729*	*0.3049*
**Fruit**			
T1	24.2 ± 20.69	7.9 ± 1.52	22.2 ± 19.40
T2	25.5 ± 23.32	7.4 ± 1.78	20.2 ± 21.81
T3	20.1 ± 19.01	6.4 ± 2.33	22.4 ± 21.04
*P*-value/χ^2^	*0.458*	***0.031***	*0.591*
**Vegetables**			
T1	21.3 ± 19.03	7.7 ± 1.55	23.0 ± 23.34
T2	22.4 ± 21.44	7.7 ± 1.76	15.8 ± 14.20
T3	26.1 ± 22.68	6.4 ± 2.33	26.0 ± 22.24
*P*-value/χ^2^	*0.598*	***0.030***	*0.318*
**Oil**			
T1	19.7 ± 20.05	7.7 ± 1.96	15.7 ± 15.69
T2	25.4 ± 24.10	7.3 ± 1.82	21.6 ± 21.67
T3	25.3 ± 19.03	6.8 ± 2.12	28.2 ± 22.82
*P*-value/χ^2^	*0.253*	*0.236*	*0.165*
**Legumes**			
T1	16.0 ± 14.57	7.0 ± 2.42	18.7 ± 16.18
T2	32.6 ± 24.76	7.1 ± 1.78	27.7 ± 24.08
T3	21.2 ± 19.38	7.7 ± 1.66	18.4 ± 19.95
*P*-value/χ^2^	***0.007***	*0.389*	*0.232*
**Nuts and seeds**			
T1	26.0 ± 26.40	7.5 ± 1.50	20.5 ± 20.21
T2	22.6 ± 15.98	7.3 ± 2.22	19.3 ± 19.15
T3	20.6 ± 17.50	7.0 ± 2.29	25.1 ± 22.41
*P*-value/χ^2^	*0.799*	*0.763*	*0.540*
**Cereals**			
T1	23.5 ± 18.66	6.7 ± 2.35	24.8 ± 22.34
T2	21.2 ± 18.95	7.4 ± 1.75	17.1 ± 15.92
T3	25.2 ± 25.37	7.6 ± 1.73	23.1 ± 22.80
*P*-value/χ^2^	*0.783*	*0.182*	*0.496*
Olive oil*			
Zero	22.1 ± 22.03	7.5 ± 1.75	19.4 ± 19.45
None-zero	27.4 ± 16.58	6.2 ± 2.43	29.4 ± 22.90
*P*-value/χ^2^	*0.060*	***0.012***	*0.078*
**Total**			
T1	24,. ± 21.13	7.3 ± 2.01	17.5 ± 16.56
T2	21.5 ± 21.30	6.9 ± 1.99	24.0 ± 20.34
T3	23.7 ± 21.08	7.5 ± 1.98	23.3 ± 24.23
*P*-value/χ^2^	*0.818*	***0.048***	*0.344*

*T, Tertile. Statistically significant values (*p* < 0.05) were given in bold.

The quantities (mg/day) of participants’ intake of phenolic compounds are presented in Table 3. Total polyphenol intake was 3,105 ± 1,879 mg/day and the majority of the total intake (1,544 ± 1,360 mg/day) was from soft drinks. A large portion of the total polyphenol intake from soft drinks was observed to be obtained from black tea (1,460 ± 1,244 mg/day). Other food groups, which contributed to the polyphenol intake, included fruits (415.4 ± 982 mg/day), pulses (381 ± 517 mg/day), and vegetables (266 ± 324 mg/day), respectively.

**TABLE 3:** Participants’ intake of phenolic compounds (mg/day) according to the food groups.

Polyphenolic compounds	Non-alcoholic beverages	Black tea	Coffee and cocoa	Fruit	Vegetables	Oil	Legumes	Nuts and seeds	Cereals	Olive oil	Total
	
	Mean ± SD	Mean ± SD	Mean ± SD	Mean ± SD	Mean ± SD	Mean ± SD	Mean ± SD	Mean ± SD	Mean ± SD	Mean ± SD	Mean ± SD
Total phenols	1,544 ± 1,360	1,460 ± 1,244	127 ± 580	415 ± 982	266 ± 324	0.5 ± 0.73	381 ± 517	180 ± 374	188 ± 255	0.3 ± 0.74	3,105 ± 1,879
Total flavonoid	568 ± 486	567 ± 484	13.7 ± 74.21	33.0 ± 50.37	68.7 ± 107.41	0.002 ± 0.005	2.8 ± 10.42	246 ± 1,137	112 ± 171	0.002 ± 0.005	1,031 ± 1,309
*Anthocyanin*	–	–	–	2.0 ± 6.40	16.1 ± 35.10	–	–	0.4 ± 0.76	6.7 ± 46.6	–	25.2 ± 58.62
*Dihydroflavanol*	–	–	0.1 ± 1.07	0.01 ± 0.18	0.002 ± 0.02	–	–	–	–	–	0.1 ± 1.09
*Flavanol*	552 ± 465	549 ± 467	12.5 ± 73.47	4.2 ± 6.00	1.0 ± 4.38	–	0.4 ± 1.71	0.4 ± 0.68	10.9 ± 52.85	–	569 ± 463
*Flavanone*	0.5 ± 4.1	–	–	15.8 ± 35.44	1.4 ± 2.79	–	–	0.008 ± 0.042	–	–	17.7 ± 35.85
*Flavones*	0.001 ± 0.008	–	0.14 ± 1.11	0.1 ± 0.32	8.1 ± 9.49	0.002 ± 0.005	0.05 ± 0.52	0.05 ± 0.50	8.3 ± 37.15	0.002 ± 0.005	16.5 ± 38.79
*Flavonol*	19.7 ± 16.4	19.4 ± 16.54	0.029 ± 0.27	2.0 ± 3.93	15.5 ± 15.0	–	2.4 ± 9.27	0.05 ± 0.21	0.05 ± 0.20	–	39.2 ± 24.48
Lignans	–	–	–	2.6 ± 6.08	0.03 ± 0.131	0.049 ± 0.119	–	6.0 ± 22.60	0.08 ± 0.08	0.05 ± 0.11	8.7 ± 24.16
Other polyphenols	–	–	0.087 ± 0.04	0.053 ± 0.47	33.613 ± 34.41	0.502 ± 1.24	0.006 ± 0.05	0.005 ± 0.051	6.01 ± 19.48	0.52 ± 1.26	39.8 ± 41.12
Total phenolic acids	0.7 ± 3.18	0.1 ± 1.32	1.1 ± 7.41	3.3 ± 16.18	25.4 ± 22.61	–	1.1 ± 7.57	39.6 ± 100.48	34.2 ± 82.83	–	105.3 ± 131.66
Stilbenes	–	–	0.005 ± 0.04	0.009 ± 0.05	–	–	0.003 ± 0.008	0.01 ± 0.055	–	–	1.1 ± 3.36

The correlations of the phytochemical and polyphenol intake and the diet quality with the disability, severity, and duration of migraine are presented in Table 4. VAS scores were significantly and inversely correlated with the PI and HEI-2015 scores (*r* = −0.37, *p* = 0.0003 and *r* = −0.37, *p* = 0.0003, respectively). The intake of phytochemicals and polyphenols and the diet quality were not correlated with migraine duration (*p* > 0.05). Regarding migraine disability, a negative and significant correlation was observed between the fruit flavanol intake (*r* = −0.21, *p* = 0.04) and disability due to migraine. Of the phenolic components, the flavanone intake (*r* = −0.27, *p* = 0.01) and the lignan intake (*r* = −0.27, *p* = 0.01) were significantly and inversely correlated with VAS scores. With respect to the food groups; migraine severity was inversely correlated with the total phenol intake from olive oil, oil, and fruits (*r* = −0.26, *p* = 0.01; *r* = −0.21, *p* = 0.04; *r* = −0.24, *p* = 0.02, respectively), and the flavonoid intake from olive oil, oil, fruits, and vegetables (*r* = −0.26, *p* = 0.01; *r* = −0.26, *p* = 0.01; *r* = −0.35, *p* = 0.0007; *r* = −0.22, *p* = 0.04, respectively). Strikingly, the intake of fruit flavanone was associated with lower migraine severity (*r* = −0.39, *p* = 0.0002).

**TABLE 4:** Correlation of phytochemical and polyphenol intake and diet quality with migraine disability, severity, and duration.

	MIDAS score		VAS score		Duration	
			
	*r*	*P*-value	*r*	*P*-value	*r*	*P*-value
Phytochemical index	0.1	0.33	–0.37	**0.0003**	0.09	0.4
HEI-2015 score	0.07	0.53	–0.37	**0.0003**	0.13	0.23
Total phenols	0.00	0.99	0.02	0.83	0.03	0.81
Total flavonoid	0.16	0.13	–0.06	0.6	0.11	0.32
*Anthocyanin*	0.06	0.59	–0.09	0.41	0.04	0.69
*Dihydroflavanol*	0.1	0.33	–0.02	0.85	–0.01	0.96
*Flavanol*	0.11	0.29	–0.06	0.59	0.04	0.7
*Flavanone*	–0.04	0.7	–0.27	**0.01**	0.04	0.69
*Flavones*	0.01	0.93	–0.17	0.11	0.04	0.69
*Flavonol*	0.08	0.45	–0.13	0.21	0.15	0.15
Lignans	–0.02	0.87	–0.27	**0.01**	0.14	0.18
Other polyphenols	0.07	0.5	0	0.99	0.05	0.64
Total phenolic acids	0.08	0.47	–0.15	0.16	0.02	0.87
Hydroxybenzoic acid	0.1	0.36	–0.16	0.12	0.02	0.84
Hydroxycinnamic acid	–0.01	0.9	–0.05	0.62	0.07	0.48
Hydroxyphenylacetic acid	0.05	0.66	–0.04	0.69	0.05	0.63
Hydroxyphenylpropionic acid	0.05	0.67	–0.06	0.58	0.02	0.87
Stilbenes	0.14	0.18	0.04	0.68	–0.03	0.78
**Total phenols^a^**						
Olive oil	0.18	0.09	–0.26	**0.01**	0.18	0.09
Oil	0.19	0.06	–0.21	**0.04**	0.15	0.17
Fruit	–0.14	0.2	–0.24	**0.02**	0	0.99
**Total flavonoid^a^**						
Oil	0.18	0.09	–0.26	**0.01**	0.18	0.09
Olive oil	0.18	0.09	–0.26	**0.01**	0.18	0.09
Fruit	–0.1	0.36	–0.35	**0.0007**	0.04	0.69
Vegetables	0.07	0.48	–0.22	**0.04**	0.21	0.06
*Flavanol*						
Fruit	–0.21	**0.04**	–0.08	0.48	–0.05	0.66
*Flavanone*						
Fruit	–0.11	0.29	–0.39	**0.0002**	0.12	0.24
*Flavones*						
Oil	0.18	0.09	–0.26	**0.01**	0.18	0.09
Vegetables	0.04	0.68	–0.2	**0.05**	0.09	0.38
Olive oil	0.18	0.09	–0.26	**0.01**	0.18	0.09
*Flavonol*						
Fruit	–0.14	0.2	–0.26	**0.01**	–0.03	0.79
**Lignans^a^**						
Oil	0.18	0.09	–0.26	**0.01**	0.18	0.09
Olive oil	0.18	0.09	–0.26	**0.01**	0.18	0.09
**Other polyphenols^a^**						
Oil	0.19	0.06	–0.26	**0.01**	0.19	0.07
Olive oil	0.18	0.09	–0.26	**0.01**	0.18	0.09

Statistically significant values (*p* < 0.05) were given in bold. ^a^Statistically significant values were given among all food groups.

Fruit flavanones, fruit flavonols, and vegetable flavonols were negatively correlated with VAS scores (est:−0.015, *p* = 0.0015; est: −0.138, *p* = 0.0002; est: −0.034, *p* = 0.0008) In the model of migraine duration, only the body-water ratio was found to be negatively correlated. A one-percent increase in the body-water ratio resulted in a 1.5 h decrease in the duration of migraines. The HEI subscores of legumes and the HEI subscores of dairy products were negatively correlated with VAS scores (est: −0.269, *p* = 0.0003; est: −0.287, 0.0002) (Table 5).

**TABLE 5:** Regression of phytochemical and polyphenol intake and diet quality with migraine disability, severity, and duration.

	est	se	*p*
**VAS**			
Intercept	–9.898	0.372	<0.0001
HEI-*Greens and beans score*	–0.269	0.071	0.0003
HEI-*Dairy score*	–0.287	0.073	0.0002
Fruit-*Flavanone*	–0.015	0.004	0.0015
Fruit-*Flavonol*	–0.138	0.036	0.0002
Vegetables-*Flavonol*	–0.034	0.01	0.0008
**Migraine duration**			
Intercept	90.643	19.337	<0.0001
Body water percentage (%)	–1.467	0.41	0.0006

## Discussion

The adoption of healthy eating behavior comes to the fore as a strategy in migraine management (7, 10). Patients’ general health status can be improved through dietary management during migraine treatment (10). It has been found that the diet quality of migraine patients is poor (10) and that poor diet quality is significantly associated with migraine (19). A recent study (*n* = 3,069) reported that healthy women with normal body weight had higher diet quality (HEI-2005 scores: 52.5 ± 0.9 and 45.9 ± 1.0, *p* < 0.05) compared to women suffering from migraine (10). Hajjarzadeh et al. (19) reported a negative and significant correlation between chronic migraine and HEI-2015 scores in women with migraine (*n* = 285) (*p* < 0.05). In parallel with the literature, in our study, a negative correlation was found between the total HEI-2015 scores and VAS scores and the examination by subcomponents revealed the significant and negative correlation of the sub-component scores of legumes and dairy products. According to HEI-2015, better diet quality includes higher consumption of total fruits, whole fruits, total vegetables, greens, and beans, whole grains, dairy, total protein foods, seafood and plant proteins, fatty acids and lower consumption of sodium, refined grains, added sugars, saturated fats (21). A good diet quality contains all nutritional components necessary for the normal maintenance of neuronal activity (22) and it may be associated with lower migraine severity. A recent study about the dietary intake of migraine patients reported that individuals with migraine had higher proinflammatory food intake and lower diet quality compared to controls (1.0 vs. 1.7, *p* = 0.02). However, a statistically significant relationship was not found between diet characteristics and migraine severity (20). In their study investigating the relationship between anti-inflammatory dietary features and migraine prognosis (*n* = 268). Ghoreishy et al. (23) found that those with the highest level of proinflammatory diet characteristics had a higher risk of suffering from severe headaches compared to those with the lowest level of proinflammatory diet characteristics (OR = 2.25%; 95% CI) and that anti-inflammatory dietary properties were inversely correlated with severe and frequent migraine attacks (24). This may result from the fact that a good-quality diet contains all nutritional components necessary for the normal maintenance of neuronal activity and is associated with lower proinflammatory properties (21). Although the mechanism of the migraine attack process has not been clarified yet, the role of inflammation is suggested (24). It is believed that the oxidant-antioxidant balance is involved in the etiopathogenesis of migraine (24). It has been suggested that the brain can cause migraine attacks to start as a homeostatic and neuroprotective response to oxidative stress (22). It is thought that healthy eating behavior and good diet quality have mitigating effects through various mechanisms on pain transmission pathways through the sufficient supply of antioxidant components. The association of migraine signs and symptoms with good diet quality may be because a good-quality diet contains antioxidants, unsaturated fatty acids, and fiber in significant amounts. The high dietary antioxidant content can reduce oxidative stress and prevent the triggering of a migraine or alleviate symptoms. Furthermore, the high quantities of unsaturated fatty acids in a good-quality diet can mitigate the induction of pain by preventing neuroinflammation or inhibiting the excessive release of inflammatory mediators. The management of the diet quality may be a good strategy to improve the prognosis of migraine, independently from changes in body weight (21). Because the relationship between diet quality and migraine has recently been introduced, the number of available studies is not adequate. Therefore, further studies are warranted to examine this relationship. Moreover, the assessment of the diet quality of migraine patients is important to alleviate/prevent symptoms.

Adequate fruit and vegetable intake may play a preventive role against increases in the incidences of inflammatory diseases. Antioxidant intake from foods prevents oxidative stress by inhibiting the initiation, propagation, and progression of the oxidative chain reaction. The natural sources of antioxidants are becoming increasingly important because of the ability of natural antioxidants to scavenge free radicals, quench molecular oxygen, and act as reducing agents in oxidative reactions (9, 25). The most common plant antioxidants with such effects are carotenoids and polyphenols (phenolic acid, anthocyanins, lignans, flavonoids, and phenolic acid) (9). Results of our study confirm that the total dietary intake of flavanones and lignans, and the intake of polyphenols, flavonoids, flavanones, and flavonols from fruits are associated with low migraine severity. Similarly, the negative correlation of VAS scores with the intake of flavonoids and flavonols from vegetables, and the negative correlation of migraine disability with the flavanol intake from fruits stress the importance of vegetables and fruits as a food group in the alleviation of migraine severity. In a study evaluating the relationship between dietary habits and primary headache (*n* = 83,214), higher fruit intake was associated with a 30% lower likelihood of suffering from primary headaches (OR: 0.70, 95% CI). Furthermore, individuals with the highest amounts of vegetable intake were found to be 16% less likely to have a primary headache than others (OR: 0.84, 95% CI) (24). Another study (*n* = 100) reported more frequent consumption of herbal beverages in individuals with migraine of moderate severity compared to those with severe migraine (*p* = 0.014) (26). In a case-control study examining the oxidant/antioxidant balance and migraine characteristics (*n* = 44), the total non-enzymatic antioxidant capacity was found to be reduced in chronic migraine patients and a negative and significant correlation was reported between the number of days with headaches in a month and the values of the following variables including catalase antioxidant enzymes (*r* = −0.60, *p* < 0.001), superoxide dismutase (*r* = −0.50, *p* < 0.001), and the Trolox equivalent antioxidant capacity (*r* = −0.61, *p* < 0.001) (27). Herbs, fruits, and vegetables can reduce the likelihood of primary headaches by preventing inflammation because they contain high levels of antioxidants and phytochemical components with anti-inflammatory effects (24). Despite the potential beneficial effects of fruit and vegetable intake on the nervous system, no studies are available, where the relationship between migraine attacks and the intake of dietary polyphenols from fruits and vegetables was examined. This relationship should be examined in depth for clarification.

Phytochemicals containing bioactive molecules with antioxidant and anti-inflammatory properties are very important in the prevention of neurodegenerative diseases (28, 29). Phytochemicals have been associated with a variety of biological activities including antioxidant and anti-inflammatory properties and antiallergic, antiviral, antiproliferative, and anticarcinogenic effects (29). Polyphenols can reduce neuroinflammation and induce cell proliferation and adult-onset neurogenesis in the hippocampus (28). Studies have proven that the beneficial effects of the Mediterranean diet in preventing neurodegeneration result from its content rich in bioactive compounds, phytochemicals, and phenols (28–31). Furthermore, the daily intake of extra virgin olive oil in quantities of 25–50 g/day in the Mediterranean diet suggests that olive oil phenols may be responsible for the potential benefits to some extent (29). In a study investigating the relationship between the characteristics of the Mediterranean diet and migraine, it was found that individuals adopting the properties of the Mediterranean diet less than others had more severe migraine disability and more severe and frequent migraine attacks (*p* < 0.05). Moreover, a significant and negative correlation was found between the scores of adherence to the Mediterranean diet and migraine severity (*r* = −0.733, *p* < 0.05) (32). Similarly, Arab et al. (33) showed that the frequency (β = −2.30, 95% CI) and duration (β = −0.42, 95%) of headaches, and migraine headache index scores (β = −47.44, 95% CI) were negatively correlated with adherence to the Mediterranean diet (33). In our study, too, we found a negative and significant correlation between migraine severity and good diet quality, high intake of phytochemicals, and the intake of polyphenols and flavonoids from olive oil and overall oil in total. Phytochemicals and olive oil polyphenols, which are the essential components of the Mediterranean diet, can reduce the induction of pain by preventing neuroinflammation and inhibiting the excessive release of inflammatory mediators. However, further comprehensive studies are needed because of the lack of an adequate number of studies in the literature.

The results of this study are important for demonstrating for the first time in the literature that migraine characteristics were correlated with diet quality and polyphenol and phytochemical intake. Despite the striking results of the study, there are some limitations. One of such limitations is the lack of investigation of the direct effects of polyphenols and phytochemicals on migraine prognosis. The effect of a diet therapy adjusted for polyphenols, phytochemicals on migraine characteristics should be investigated. Another limitation is that the study included a limited number of subjects and examined only episodic migraine patients. There is a need for detailed information about the possible relationship in migraine types other than episodic migraine patients. In addition, assessments of patients were subjective based on the perceived severity of pain and the ability to recall. Likewise, another limitation of the study is that the dietary patterns were assessed based on patients’ statements. Comprehensive studies are warranted to analyze different age groups, genders, and migraine types. A detailed dietary assessment strategy combined with medical treatment provide a holistic assessment of migraine. Examining the diets of migraine patients, especially polyphenol and phytochemical intakes may help to reveal the migraine-polyphenol-phytochemical relationship.

## Conclusion

The results of this study reveal that adopting a high-quality diet and higher total phytochemical and polyphenols (flavanones and lignans) intake, especially vegetable oil, olive oil, fruit, and vegetable phenols and flavonoids is associated with lower migraine severity. Nevertheless, more high-quality prospective studies are needed to clarify the causal relationship between them.

## Data availability statement

The raw data supporting the conclusions of this article will be made available by the authors, without undue reservation.

## Ethics statement

The studies involving human participants were reviewed and approved by the Istanbul Medipol University Non-Interventional Clinical Research Ethics Committee on March 18, 2021, with decision number 303. The patients/participants provided their written informed consent to participate in this study.

## Author contributions

HB and TU contributed to the conception and design of the study. HB collected article data. HB and MP analyzed all survey data. MK contributed to all statistical analyses and interpreted data. HB, MP, and TU wrote the manuscript. All authors contributed to the article and approved the submitted version.

## References

[1] Kıvrak Y Özen cS Yücel Y. Migren ve gerilim başağrısı olan hastalarda anksiyete ve umutsuzluk düzeyleri [Anxiety and hopelessness levels in patients with migraine and tension headache]. *Dicle Med J.* (2009) 36:173–7.

[2] Galletti F Cupini LM Corbelli I Calabresi P Sarchielli P. Pathophysiological basis of migraine prophylaxis. *Prog Neurobiol.* (2009) 89:176–92. 10.1016/j.pneurobio.2009.07.005 19654035

[3] World Health Organisation [WHO]. *The World Health Report 2001 - Mental Health: New Understanding, New Hope.* (2001). Available online at: https://apps.who.int/iris/handle/10665/42390 (accessed on Sep 9, 2022).

[4] Yücel Y. Migren baş ağrısında tanı ve tedavi yaklaşımları [Diagnosis and treatment approaches in migraine headache]. *Dicle Med J.* (2008) 35:281–6.

[5] Ayalp S Sahin Ş Benli Aksungar F Karsj̧dag S. Aurasiz migrende trombosit serotonin düzeylerinin değerlendirilmesi [Evaluation of platelet serotonin levels in migraine without aura]. *Agrı.* (2012) 24:117–22. 10.5505/agri.2012.74946 22865518

[6] Siva A. Başağrısı epidemiyolojisi [Epidemiology of headache]. *Türkiye Klin Nöroloji Derg.* (2003) 1:94–7.

[7] Mirzababaei A Khorsha F Togha M Yekaninejad MS Okhovat AA Mirzaei K. Associations between adherence to dietary approaches to stop hypertension (DASH) diet and migraine headache severity and duration among women. *Nutr Neurosci.* (2020) 23:335–42. 10.1080/1028415X.2018.1503848 30064351

[8] Valentina AA de Edelenyi FS Druesne-Pecollo N Touvier M Hercberg S Galan P. Macronutrient intake in relation to migraine and non-migraine headaches. *Nutrients.* (2018) 10:1309. 10.3390/nu10091309 30223543 PMC6164759

[9] Goschorska M Gutowska I Baranowska-bosiacka I Barczak K Chlubek D. The use of antioxidants in the treatment of migraine. *Antioxidants.* (2020) 9:116. 10.3390/antiox9020116 32012936 PMC7070237

[10] Evans EW Lipton RB Peterlin BL Raynor HA Thomas JG O’Leary KC Dietary intake patterns and diet quality in a nationally representative sample of women with and without severe headache or migraine. *Headache.* (2015) 55:550–61. 10.1111/head.12527 25758250 PMC4443434

[11] Özön AÖ Karadaş Ö Özge A. Efficacy of diet restriction on migraines. *Noro Psikiyatr Ars.* (2018) 55:233–7. 10.5152/npa.2016.15961 30224869 PMC6138234

[12] Pogoda JM Gross NB Arakaki X Fonteh AN Cowan RP Harrington MG. Severe headache or migraine history is inversely correlated with dietary sodium intake: NHANES 1999-2004. *Headache.* (2016) 56:688–98. 10.1111/head.12792 27016121 PMC4836999

[13] Rist PM Buring JE Kurth T. Dietary patterns according to headache and migraine status: a cross-sectional study. *Cephalalgia.* (2015) 35:767–75. 10.1177/0333102414560634 25424709 PMC4442763

[14] Arslan M Albaş S Küçükerdem H Pamuk G Can H. The evaluation of pain treatment the effectiveness in palliative cancer patients with visual analog scale. *Fam Pract Palliat Care.* (2016) 1:5–8. 10.22391/920.182939

[15] Kılıç S Derman O Akgül S Kanbur N Kutluk T Aysun S. The use of the MIDAS questionnaire to assess migraine and tension type headache in adolescents. *Turkiye Klin J Med Sci.* (2012) 32:466–71. 10.5336/medsci.2011-25330

[16] Krebs-Smith SM Pannucci TE Subar AF Kirkpatrick SI Lerman JL Tooze JA Update of the healthy eating index: HEI-2015. *J Acad Nutr Diet.* (2018) 118:1591–602. 10.1016/j.jand.2018.05.021 30146071 PMC6719291

[17] McCarty MF. Proposal for a dietary “phytochemical index”. *Med Hypotheses.* (2004) 63:813–7. 10.1016/j.mehy.2002.11.004 15488652

[18] Haytowitz DB Wu X Bhagwat S. *USDA Database for the Flavonoid Content of Selected Foods, Release 3.3.* Maryland: U.S. Department of Agriculture, Agricultural Research Service (2018).

[19] Hajjarzadeh S Nikniaz Z Shalilahmadi D Mahdavi R Behrouz M. Comparison of diet quality between women with chronic and episodic migraine. *Headache.* (2019) 59:1221–8. 10.1111/head.13623 31453643

[20] Martins LB Braga Tibães JR Dos Santos Rodrigues AM Hassanzadeh Keshteli A Karam Vono C Borges E Borges J The quality and inflammatory index of the diet of patients with migraine. *Nutr Neurosci.* (2021) 25:1–8. 10.1080/1028415X.2021.1939935 34148510

[21] Costa A Rodrigues A Martins L Santos L Gomez R Teixeira A. Nutritional intervention may improve migraine severity: a pilot study. *Arq Neuropsiquiatr.* (2019) 77:723–30. 10.1590/0004-282x20190121 31664348

[22] Borkum JM. The migraine attack as a homeostatic, neuroprotective response to brain oxidative stress: preliminary evidence for a theory. *Headache.* (2018) 58:118–35. 10.1111/head.13214 29034461

[23] Ghoreishy SM Askari G Mohammadi H Campbell MS Khorvash F Arab A. Associations between potential inflammatory properties of the diet and frequency, duration, and severity of migraine headaches: a cross-sectional study. *Sci Rep.* (2022) 12:2878. 10.1038/s41598-022-06819-y 35190593 PMC8861209

[24] Mansouri M Sharifi F Varmaghani M Shokri A Rahdar H Keshtkar A Fruit and vegetable consumption in relation to primary headaches: the MEPHASOUS study. *Eat Weight Disord.* (2021) 26:1617–26. 10.1007/s40519-020-00984-7 32789621

[25] Baiano A del Nobile MA. Antioxidant compounds from vegetable matrices: biosynthesis, occurrence, and extraction systems. *Crit Rev Food Sci Nutr.* (2015) 56:2053–68. 10.1080/10408398.2013.812059 25751787

[26] Magdy R Eid RA Hassan M Abdelghaffar M El Sayed AF Mohammed Z The potential impact of nutritional intake on symptoms severity in patients with comorbid migraine and irritable bowel syndrome. *BMC Neurol.* (2022) 22:199. 10.1186/s12883-022-02723-0 35637446 PMC9150376

[27] Togha M Razeghi Jahromi S Ghorbani Z Ghaemi A Rafiee P. An investigation of oxidant/antioxidant balance in patients with migraine: a case-control study. *BMC Neurol.* (2019) 19:323. 10.1186/s12883-019-1555-4 31837702 PMC6911287

[28] Kaplan A Zelicha H Yaskolka Meir A Rinott E Tsaban G Levakov G The effect of a high-polyphenol Mediterranean diet (Green-MED) combined with physical activity on age-related brain atrophy: the dietary intervention randomized controlled trial polyphenols unprocessed study (DIRECT PLUS). *Am J Clin Nutr.* (2022) 115:1270–81. 10.1093/ajcn/nqac001 35021194 PMC9071484

[29] Angeloni C Malaguti M Barbalace MC Hrelia S. Bioactivity of olive oil phenols in neuroprotection. *Int J Mol Sci.* (2017) 18:2230. 10.3390/ijms18112230 29068387 PMC5713200

[30] Cannataro R Fazio A La Torre C Caroleo MC Cione E. Polyphenols in the mediterranean diet: from dietary sources to microRNA modulation. *Antioxidants.* (2021) 10:328. 10.3390/antiox10020328 33672251 PMC7926722

[31] Hernández JM Rentero MPZ. Bioactive compounds contained in Mediterranean Diet and their effects on neurodegenerative diseases. In: Shiomi N editor. *Current Topics on Superfoods.* London: IntechOpen (2018). p. 13–32. 10.5772/intechopen.74084

[32] Bakırhan H Yıldıran H Uyar Cankay T. Associations between diet quality, DASH and Mediterranean dietary patterns and migraine characteristics. *Nutr Neurosci.* (2021) 11:1–11. 10.1080/1028415X.2021.1963065 34379573

[33] Arab A Khorvash F Karimi E Hadi A Askari G. Associations between adherence to Mediterranean dietary pattern and frequency, duration, and severity of migraine headache: a cross-sectional study. *Nutr Neurosci.* (2021) 1–10. 10.1080/1028415X.2021.2009162 [Epub ahead of print]. 34870564

